# Association of NT-proBNP and sST2 with Diastolic Dysfunction in Cirrhotic Patients and Its Therapeutic Implications

**DOI:** 10.3390/ijms27010261

**Published:** 2025-12-26

**Authors:** Roxana Mihaela Chiorescu, Alexandru Ruda, Romeo Chira, Georgiana Nagy, Adriana Bințințan, Ștefan Chiorescu, Mihaela Mocan

**Affiliations:** 1 Internal Medicine Department, “Iuliu Hatieganu” University of Medicine and Pharmacy, 400012 Cluj-Napoca, Romania; roxana.chiorescu11@gmail.com (R.M.C.); romeochira@yahoo.com (R.C.); georgiana.nagy@umfcluj.ro (G.N.); mihaela.mocan@gmail.com (M.M.); 2 Department of Internal Medicine, Emergency Clinical County Hospital, 400006 Cluj-Napoca, Romania; 3 Department of Cardiology, Nicolae Stăncioiu Heart Institute, 400001 Cluj-Napoca, Romania; 4 Department of Gastroenterology, Emergency Clinical County Hospital, 400006 Cluj-Napoca, Romania; abintintan@yahoo.com; 5 Department of Surgery, “Iuliu Hatieganu” University of Medicine and Pharmacy, 400012 Cluj-Napoca, Romania; stefanc74@yahoo.com; 6 Department of Surgery, Emergency Clinical County Hospital, 400006 Cluj-Napoca, Romania

**Keywords:** cirrhotic cardiomyopathy, cardiac diastolic function, NT-proBNP, sST2

## Abstract

Cirrhotic cardiomyopathy encompasses structural and functional cardiac abnormalities occurring in patients with liver cirrhosis despite the absence of pre-existing heart disease, yet its diagnosis remains challenging. Although echocardiography is the standard diagnostic tool, circulating biomarkers may provide complementary value when imaging findings are inconclusive. This study evaluated the association between N-terminal pro-B-type natriuretic Peptide (NT-proBNP), soluble Suppression of Tumorigenicity 2 (sST2), and diastolic dysfunction in cirrhotic patients without known cardiac disease. We conducted a prospective case–control study including 83 participants (43 patients with non-alcoholic cirrhosis and 40 healthy controls), assessed clinically, biochemically, and echocardiographically between June 2020 and July 2021. Cirrhotic patients showed significantly higher NT-proBNP (94.17 ± 151.36 pg/mL vs. 19.2 ± 5.47 pg/mL, *p* < 0.001) and sST2 levels (5.4 ± 2.31 ng/mL vs. 2.4 ± 0.99 ng/mL, *p* < 0.001). NT-proBNP demonstrated limited diagnostic accuracy for diastolic dysfunction (accuracy 52.6%, sensitivity 50%, specificity 60%, AUC 0.51), but it correlated modestly with congestion markers such as left atrial volume and pulmonary artery systolic pressure. A multimarker model combining age, NT-proBNP, and sST2 substantially improved diagnostic performance for diastolic dysfunction (accuracy 75%, sensitivity 77.1%, specificity 71.4%, AUC 0.925). In conclusion, NT-proBNP is associated with diastolic dysfunction but is influenced by cirrhosis congestion status. A combined NT-proBNP and sST2 assessment enhances diagnostic precision and may aid therapeutic decision-making, particularly regarding congestion and diuretic management in cirrhotic patients.

## 1. Introduction

Liver cirrhosis represents the terminal stage of a wide spectrum of chronic liver diseases and remains a major contributor to global morbidity and mortality [1]. In addition to progressive hepatic injury, cirrhosis is associated with multiple extrahepatic complications, among which cardiovascular involvement carries significant prognostic implications [2].

Cirrhotic cardiomyopathy (CCM) is known as a constellation of structural and functional cardiac abnormalities that develop in patients with cirrhosis and without other structural heart diseases The manifestations are subclinical and are identified by echocardiographical examination in the presence of left ventricular systolic and/or diastolic (LVSD/LVDD) which become particularly relevant in situations of hemodynamic stress, such as infection, hemorrhage, or liver transplantation [3].

The principal causes of liver cirrhosis continue to be chronic viral hepatitis, alcohol-associated liver disease, and metabolic dysfunction-associated steatotic liver disease [4]. Historically, alcohol was considered the primary cause of cardiac abnormalities in patients diagnosed with cirrhosis, no matter the etiology. A change in paradigm was proposed by Kowalski and Ablemann in 1953, who showed cardiac pathological changes in patients with cirrhosis independent of prior alcohol intake [5].

The pathogenesis of cirrhotic cardiomyopathy is complex and involves the interaction between hemodynamic mechanisms (circulatory hyperdynamics, congestion, activation of neurohormonal systems) [6] and inflammatory and fibrotic processes (myocardial remodeling, inflammatory infiltrates, collagen depositions, and cytokine changes) [7]. Together, these processes drive the progression of cardiac dysfunction and account for the challenges associated with early diagnosis. In recent decades, numerous pathophysiological mechanisms have been described to explain cirrhotic cardiomyopathy, yet early diagnosis remains challenging. Traditionally, it is based on echocardiographic criteria, but recent research showed that biomarkers might be of use in clinical practice as additional diagnostic and prognostic tools, especially in situations where echocardiography did not provide sufficient criteria for diagnosis or was difficult to perform. Such situations include patients who associate various comorbidities, pulmonary emphysema, atrial fibrillation, and tachycardia, among others [7,8].

Natriuretic peptides remain the main biomarkers associated with diastolic dysfunction because of their availability and low costs. Still, there is evidence that N-terminal prohormone brain natriuretic peptide (NT-proBNP) is influenced by the presence and severity of liver cirrhosis [9], resulting in lower specificity in those cohorts.

Soluble suppression of tumorigenesis-2 (sST2) is an inflammation- and fibrosis-associated biomarker that has demonstrated diagnostic relevance in the assessment of cardiac remodeling and impaired diastolic function [9] as well as prognostic value for all-cause and cardiovascular mortality and for heart failure (HF)-related hospitalization in patients with chronic HF [10].

Although natriuretic peptides (B-type Natriuretic Peptide [BNP] and NT-proBNP) are the most widely used biomarkers, their limitations in the setting of cirrhosis have underscored the need for additional diagnostic tools. Consequently, a current research direction focuses on combining congestion biomarkers (such as NT-proBNP) with markers of inflammation and fibrosis (such as sST2). This integrated approach may improve the accuracy of diagnosis and the overall management of cirrhotic cardiomyopathy.

The present study aims to evaluate the association between NT-proBNP and sST2 levels and the presence of diastolic dysfunction in patients diagnosed with non-alcoholic cirrhosis.

## 2. Results and Discussions

### 2.1. Results

The study population consisted of 83 participants, divided into two groups: patients with cirrhosis (n = 43) and healthy controls (n = 40). The mean age of the study group was 62.66 ± 7.55 years, while that of the control group was 57.6 ± 12.75 years, with no statistically significant difference between the two groups (*p* = NS). The sex distribution was balanced: 19 males and 24 females in the study group, and 22 males and 18 females in the control group. The body mass index (BMI) was comparable between groups (28.72 ± 3.67 kg/m^2^ vs. 29.39 ± 3.25 kg/m^2^, *p* = NS) (Table 1).

Clinical manifestations included: ascites in 25.6%, splenomegaly in 44%, and hepatic encephalopathy in 9.3% of the patients. The mean heart rate was 73 ± 11 beats per minute in the study group and 55 ± 12 beats per minute in the control group (*p* = NS).

Albumin levels averaged 3.72 g/dL (Standard Deviation (SD)—0.68), with values ranging from 2.32 to 4.74 g/dL. Total Bilirubin levels had a mean of 1.39 mg/dL (SD 1.11), spanning 0.35 to 4.18 mg/dL. Creatinine levels averaged 0.80 mg/dL (SD 0.33), with a range of 0.46 to 1.85 mg/dL.

According to the Child–Pugh classification, most patients were in Class A (76.35%), followed by Class B (13.15%) and Class C (10.5%). A comparison between clinical characteristics and lab test results in the analyzed groups is synthesized in Table 1.

The main echocardiographic parameters for patients with cirrhosis and for controls are presented in Table 2.

Univariate analyses were conducted to examine potential differences in NT-proBNP and sST2 levels between the patient and control groups, as well as to assess differences in the prevalence of diastolic dysfunction. The patient group had significantly higher NT-proBNP levels (median = 39.8 ng/mL, mean = 94.17 ng/mL, SD = 151.36 ng/mL) than the control group (median = 18.68, mean = 19.2, SD = 5.47), U = 1580, *p* < 0.001. A relevant difference in sST2 levels was also observed, with patients showing substantially higher values (median = 5.3 ng/mL, mean = 5.4 ng/mL SD = 2.31 ng/mL) compared with controls (median = 2.05 ng/mL, mean = 2.4 ng/mL, SD = 0.99 ng/mL), U = 1579, *p* < 0.001. No statistically significant difference was found in the prevalence of diastolic dysfunction between patients (22 individuals, 51.16%) and controls (22 individuals, 55%) (*p* = NS) (Table 2).

These results suggest that NT-proBNP and sST2 levels may be influenced by cirrhosis, particularly given that the prevalence of diastolic dysfunction did not differ significantly between groups. In both the cirrhosis and the control groups, NT-proBNP levels were strongly correlated with the presence of diastolic dysfunction. In the cirrhosis group, the Mann–Whitney test revealed a significant difference in NT-proBNP values between patients with diastolic dysfunction (U = 111.0000, *p* = 0.0036). Patients without diastolic dysfunction (n = 21) had lower NT-proBNP levels (mean = 53.20 pg/mL, median = 30.80 pg/mL) compared to those with diastolic dysfunction (n = 22), who had elevated levels (mean = 132.78 pg/mL, median = 59.10 pg/mL).

**Figure 1:**
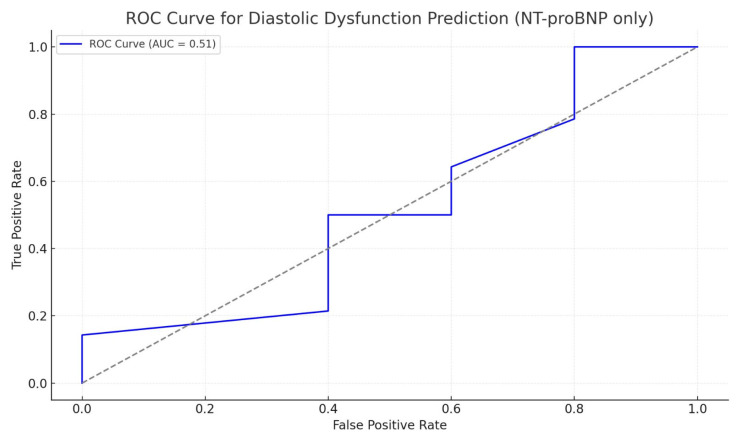
Predictive ability of NT-proBNP to differentiate patients with diastolic dysfunction.

We assessed the diagnostic performance of NT-proBNP in identifying diastolic dysfunction, using ROC curve analysis. The AUC was approximately 0.51, indicating weak diagnostic accuracy, with a sensitivity of about 50% and a specificity of roughly 60%. These results confirm that NT-proBNP has limited value as a standalone biomarker for detecting diastolic dysfunction and is more appropriately used as part of a multi-parametric diagnostic approach (Figure 1).

In the control group, the ROC curve highlighted excellent discriminative performance for NT-proBNP, with an AUC of 0.92, suggesting effective differentiation between clinical categories, with a sensitivity of ~0.82 and specificity of ~1.00, indicating high diagnostic accuracy.

In patients with cirrhosis, NT-proBNP appears to have limited utility as a single diagnostic marker, likely due to disease-specific confounding mechanisms.

Although NT-proBNP values tended to increase with worsening liver disease (higher Child-Pugh class), this association did not reach statistical significance. The Kruskal–Wallis test confirmed the absence of significant differences between Child-Pugh classes (H = 2.8184, *p* = 0.2443): class A (n = 29)—mean 70.55 pg/mL, median 39.80 pg/mL; class B (n = 5)—mean 84.72 pg/mL, median 57.60 pg/mL; class C (n = 4)—mean 368.80 pg/mL, median 304.80 pg/mL. One of the limitations of this study was the small number of patients with Child B and Child C cirrhosis.

Regarding sST2, no correlation was observed between biomarker levels and diastolic dysfunction in either group. In the cirrhosis group, the Mann–Whitney test showed no significant difference in sST2 values between patients with and without diastolic dysfunction (U = 217.0000, *p* = 0.7427). Patients without diastolic dysfunction (n = 21) had slightly lower sST2 levels (mean = 5.12 ng/mL, median = 5.50 ng/mL), compared to those with dysfunction (n = 22) (mean = 5.67 ng/mL, median = 5.30 ng/mL).

Similarly, in the control group, no significant differences were noted (U = 230, *p* = 0.3906), as values remained comparable between subjects with (n = 22) and without diastolic dysfunction (n = 18).

When analyzed sSt2 values according to liver disease severity, no significant differences were observed across Child-Pugh classes (H = 0.1604, *p* = 0.9230), although values displayed a decreasing trend: class A (n = 29)—mean 5.84 ng/mL, median 5.40 ng/mL; class B (n = 5)—mean 5.46 ng/mL, median 5.60 ng/mL; class C (n = 4)—mean 5.33 ng/mL, median 5.25 ng/mL.

NT-proBNP concentrations were significantly higher in patients with diastolic dysfunction and ascites than in those with ascites alone. Patients without diastolic dysfunction and with ascites had low NT-proBNP values (mean = 31.55 pg/mL, median = 30.80 pg/mL), whereas patients with both diastolic dysfunction and ascites had markedly elevated levels (mean = 344.54 pg/mL, median = 272.00 pg/mL). The difference was statistically significant, according to the Mann–Whitney U test (U = 0.0000, *p* = 0.0195).

The presence of ascites did not influence sST2 levels, as demonstrated by the absence of important differences between subgroups (U = 86.0000, *p* = 1.0000): without ascites (mean = 5.79 ng/mL, median = 5.40 ng/mL) versus with ascites (mean = 5.53 ng/mL, median = 5.40 ng/mL).

From a pathophysiological perspective, NT-proBNP increases primarily as a marker of cardiac congestion, whereas sST2 reflects inflammation and myocardial remodeling and correlates more strongly with systolic dysfunction. Therefore, we aimed to evaluate the relationship between NT-proBNP and sST2 levels, echocardiographic parameters, and the presence of ascites.

Because sST2 is not influenced by the severity of cirrhosis or the presence of ascites, it serves as a complementary marker to NT-proBNP in the diagnosis of diastolic dysfunction.

Concerning associations with echocardiographic parameters, NT-proBNP levels correlated moderately and significantly with left atrial volume (r = 0.3606, *p* = 0.0223) and pulmonary artery systolic pressure (PASP) (r = 0.4793, *p* = 0.0178). No significant associations were identified for sST2 (left atrium: r = 0.0576, *p* = 0.7239; PASP: r = 0.3042, *p* = 0.1485). Neither biomarker correlated with the E/e′ ratio (NT-proBNP: r = −0.0555, *p* = 0.7271; sST2: r = −0.0259, *p* = 0.8708).

No significant correlations were found with TAPSE for NT-proBNP (r = 0.0035, *p* = 0.9834).

A multivariate logistic regression analysis was conducted to assess the combined predictive value of age, ascites, Child-Pugh classification, creatinine, sST2, and NT-proBNP in predicting diastolic dysfunction. The overall model was statistically significant, χ^2^ (6) = 28.33, *p* < 0.001, indicating the reliable accuracy of the aforementioned predictors in distinguishing between the presence and absence of diastolic dysfunction. Age emerged as a borderline significant predictor (B = 0.45, SE = 0.24, *p* = 0.054), suggesting that older individuals may be more likely to exhibit diastolic dysfunction. The other variables—ascites, Child-Pugh classification, creatinine, sST2, and NT-proBNP—were not statistically significant contributors (all *p* > 0.30). The model explained 41.1% of the variance in diastolic dysfunction, indicating a moderate overall fit.

**Figure 2:**
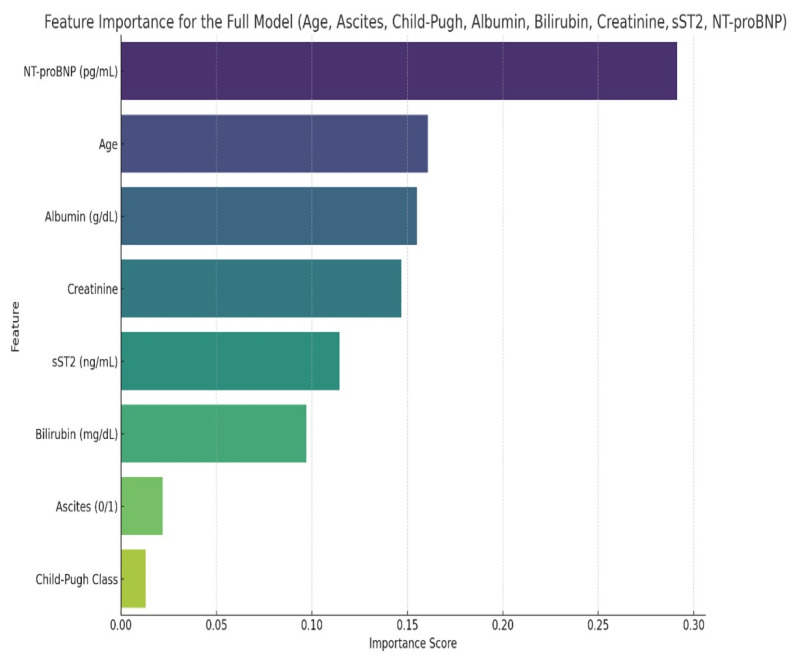
The feature importance of the initially considered factors.

A decision tree model was subsequently developed, including all relevant variables: age, ascites, Child-Pugh classification, albumin, bilirubin, creatinine, sST2, and NT-proBNP. The initial model achieved 61.3% accuracy, with 66.7% sensitivity and 55.6% specificity. Feature importance analysis identified sST2, NT-proBNP, and age as the most influential predictors (Figure 2), while ascites, Child-Pugh classification, albumin, bilirubin, and creatinine contributed minimally to predictive performance.

**Figure 3:**
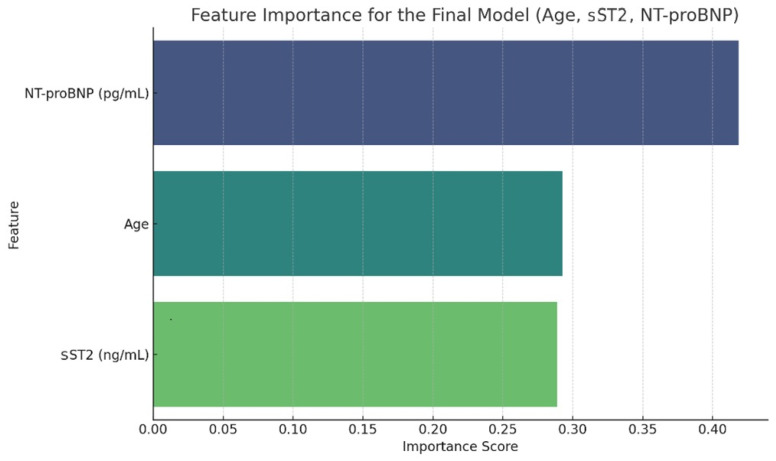
Feature importance of the final model (Age, sST2, NT-proBNP).

Based on these results, the less influential predictors were removed, yielding a refined model including only age, sST2, and NT-proBNP (Figure 3).

**Figure 4:**
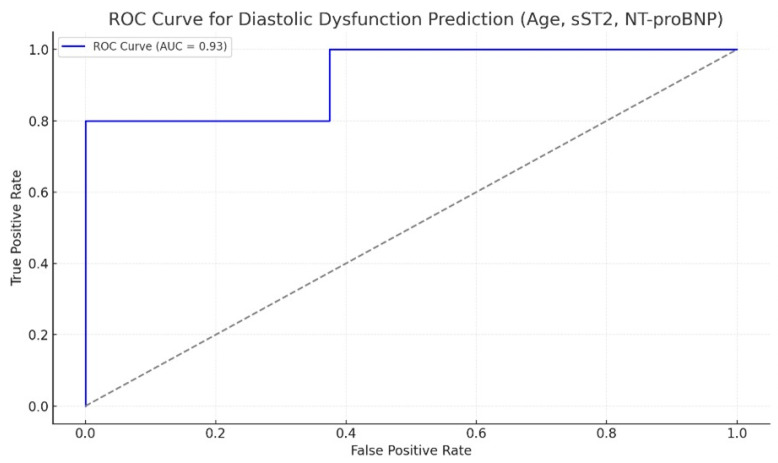
Receiver Operating Characteristic curve for the final model including age, sST2, and NT-proBNP.

The refined model demonstrated improved performance, with 75.0% accuracy, 77.1% sensitivity, and 71.4% specificity. The area under the ROC curve was 0.925, indicating excellent discriminatory ability (Figure 4).

Overall, this streamlined model offers a more parsimonious yet robust approach for predicting diastolic dysfunction.

**Figure 5:**
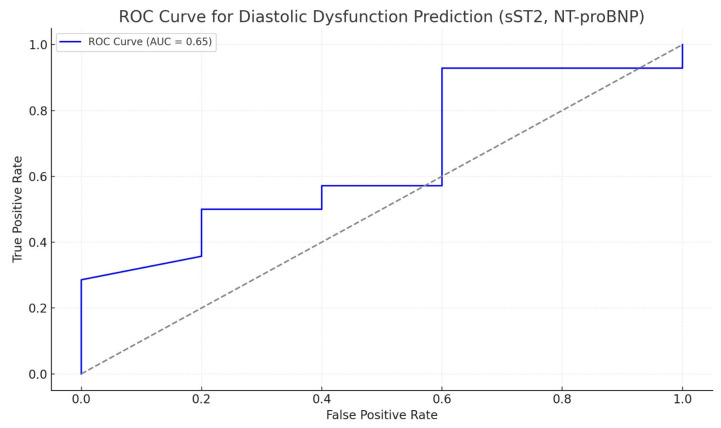
Predictive ability of NT-proBNP combined with sST2 to differentiate patients with diastolic dysfunction.

After excluding age from the predictive model for diastolic dysfunction and using only NT-proBNP and sST2, we obtained a model with slightly lower—but still acceptable—predictive performance. The resulting ROC curve showed an accuracy of 57.9%, a sensitivity of 57.1%, a specificity of 60.0%, and an AUC of 0.65 (Figure 5).

### 2.2. Discussions

#### 2.2.1. Pathophysiological Significance of NT-proBNP and sST2 in Cirrhotic Cardiomyopathy

Liver cirrhosis remains one of the major causes of morbidity and mortality around the world [11]. As a result of pathological hyperdynamic circulation and synthetic/metabolic alterations of cardiac proteins, lipids, and carbohydrates (e.g., β-adrenergic receptors, collagens, cholesterol, and lectins) [12], cirrhosis is associated with a wide spectrum of cardiovascular abnormalities [13].

Cirrhotic cardiomyopathy (CCM) is defined by the presence of diastolic dysfunction, impaired myocardial contractile responsiveness to physiological or pharmacological stress, and electrophysiological abnormalities [14].

Although patients with liver cirrhosis frequently exhibit a hyperdynamic circulatory state characterized by increased cardiac output and apparently preserved cardiac function at rest, underlying structural and functional myocardial alterations become clinically manifest under stress conditions—such as infections, invasive procedures, or liver transplantation—often resulting in adverse outcomes and poorer prognosis. [15]. Therefore, early diagnosis and appropriate management of this condition are crucial [16].

Diagnostic challenges arise from the often-silent nature of the disease and the absence of universally accepted diagnostic criteria, particularly in cases where echocardiography is technically difficult or inconclusive. Currently, no specific treatment exists for cirrhotic cardiomyopathy; therapy follows the general HF guidelines, tailored to the clinical phenotype (with or without systolic dysfunction) [17].

Novel therapeutic molecules targeting different stages of the pathophysiological processes involved in CCM are under investigation, but further studies are required before they can be implemented in clinical practice.

According to the 2020 criteria [17], cirrhotic cardiomyopathy can be diagnosed by identifying either left ventricular systolic dysfunction (LVEF < 50% or GLS < −18%) or advanced diastolic dysfunction, defined by at least three of the following: E/e′ > 14, septal e′ < 7 cm/s or lateral e′ < 10 cm/s, TR velocity > 2.8 m/s, or LAVI > 34 mL/m^2^. If only two diastolic criteria are present, the diagnosis remains probable and requires additional testing.

Complementary investigations may improve diagnostic accuracy [17]. Stress echocardiography (pharmacologic or exercise) evaluates contractile reserve, while GLS helps in detecting subclinical systolic dysfunction when not previously measured. BNP/NT-proBNP and high-sensitivity troponin can reflect myocardial stress. Cardiac MRI provides further characterization, including diffuse fibrosis on T1 mapping, particularly when echocardiographic images are suboptimal. ECG or Holter monitoring may identify arrhythmia or QTc prolongation.

When echocardiographic evaluation is difficult (e.g., patients with pulmonary emphysema, tachycardia, or atrial fibrillation) or criteria are only partially met, supportive biomarkers may aid in diagnosis. The choice of biomarkers should be guided by the underlying pathophysiological mechanisms of cirrhotic cardiomyopathy [18].

From a pathophysiological perspective, a complex bidirectional interaction exists between the heart and the liver, constituting a true cardio-hepatic vicious cycle. On one hand, HF induces hepatic venous congestion and reduced hepatic perfusion, promoting fibrotic changes and, in advanced stages, cardiogenic cirrhosis. On the other hand, cirrhosis leads to systemic vasodilation and activation of neurohormonal pathways that adversely affect cardiac function, ultimately contributing to the development of CCM.

These processes perpetuate each other: cirrhosis worsens HF, and HF accelerates hepatic deterioration, making diagnosis and treatment challenging. Both pathologies trigger a similar neurohormonal compensatory response.

In HF, reduced cardiac output leads to a decrease in effective arterial blood volume, whereas in liver cirrhosis, splanchnic vasodilation and portal hypertension generate a comparable state of “arterial underfilling”. This triggers compensatory activation of RAAS (promoting sodium and water retention and aggravating ascites), antidiuretic hormone (water retention and dilutional hyponatremia), and the SNS (vasoconstriction with further hepatorenal compromise).

Cirrhosis itself is characterized by architectural distortion, hepatocellular dysfunction, and increased portal pressure, leading to mesenteric congestion. Portal hypertension promotes gut stasis, microbial translocation, and endotoxemia, driving a systemic pro-inflammatory cytokine response [19].

Hepatocellular dysfunction promotes systemic accumulation of vasoactive mediators—including bile acids—disrupts cardiomyocyte membrane lipid composition, and alters structural proteins, such as sarcomeric filaments.

Elevated circulating bile acid concentrations directly impair myocardial contractility and promote a shift from the α- to the β-myosin heavy chain (MHC) isoform [20]. Increased serum galectin-3 levels have been associated with both systolic [21] and diastolic dysfunction [7,22]. β-adrenergic receptor dysregulation can result in systolic impairment [23], while alterations in membrane fluidity, titin abnormalities, and decreased collagen-1 (with a compensatory increase in collagen-3) have been linked to diastolic dysfunction [24,25].

**Figure 6:**
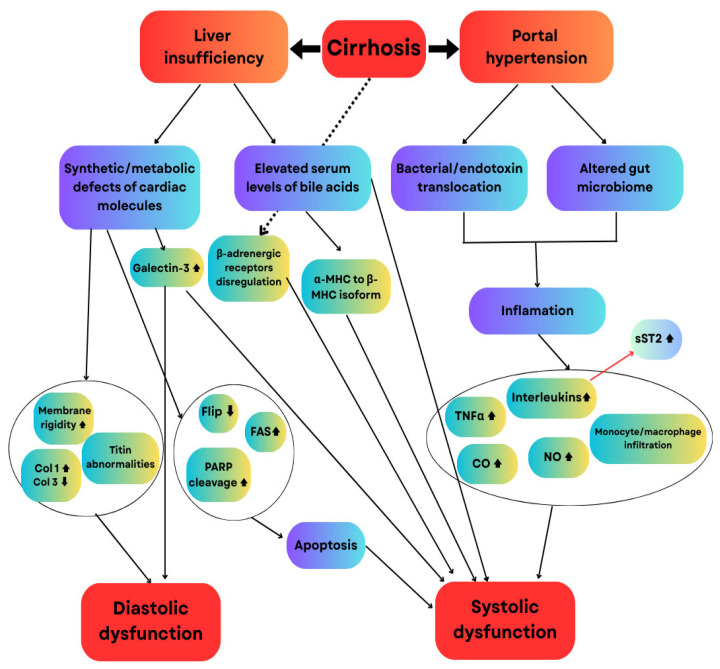
Pathophysiological mechanisms of CCM. MHC, myosin heavy chain; Col, collagen; TNF, tumor necrosis factor; CO, carbon monoxide; NO, nitric oxide; PARP, poly (ADP-ribose) polymerase; Flice-like Inhibitory Protein (FLIP), FAS-associated death domain-like interleukin 1β-converting enzyme inhibitory protein FAS. Upward arrows represent elevated concentrations, while downward arrows represent reduced concentrations. Interconnecting arrows illustrate the influence or regulatory relationships among the molecules. Adapted from [19].

Apoptosis plays a crucial role, with increased poly (ADP-ribose) polymerase (PARP) cleavage and Fas protein expression [26]. Chronic inflammation promotes macrophage infiltration [27] and increases Tumor Necrosis Factor Alpha (TNF-α) [28] and interleukin-6 [29] in cardiac tissue, leading to reduced contractility. Overproduction of nitric oxide (NO) and carbon monoxide (CO) further impairs systolic function by activating soluble guanylate cyclase to generate Cyclic Guanosine Monophosphate (cGMP) [15,30] (Figure 6).

Based on these mechanisms, we selected NT-proBNP as an indicator of circulatory alterations in cirrhosis and sST2 as an inflammatory marker influenced by cardiac remodeling.

**Figure 7:**
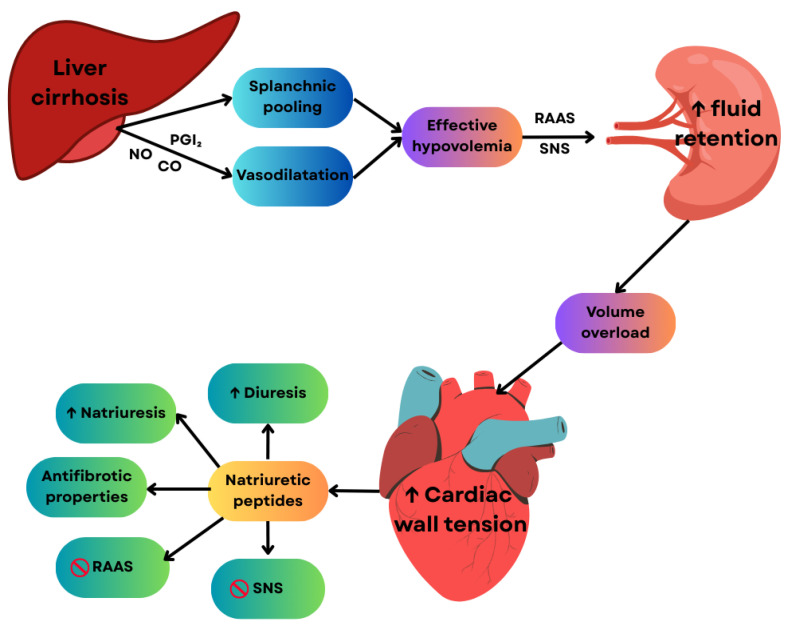
Pathophysiology of natriuretic peptides in cirrhosis. Upward arrows represent elevated concentrations. Interconnecting arrows illustrate the influence or regulatory relationships among the molecules. CO, carbon monoxide; NO, nitric oxide; PGI_2_, Prostacyclin; RAAS, Renin–Angiotensin–Aldosterone System; SNS, Sympathetic Nervous System.

Natriuretic peptides such as BNP, NT-proBNP, and mid-regional pro-atrial natriuretic peptide (MR-proANP) have been used for a long time to assess cardiac dysfunction and to diagnose and predict HF, even though cut-off values are different in diastolic dysfunction as compared to systolic dysfunction [31,32]. Plasma concentrations of NT-proBNP are markedly elevated in patients with cirrhosis and demonstrate a positive correlation with the severity of hepatic dysfunction, as stratified by the Child-Pugh classification system [33]. In cirrhosis, systemic vasodilation and splanchnic pooling lead to effective hypovolemia, triggering activation of the sympathetic nervous system and the renin–angiotensin–aldosterone system. This pathophysiological process leads to intravascular volume overload and increased myocardial wall stress, particularly at the atrial and ventricular levels, resulting in enhanced synthesis and release of natriuretic peptides [14]. These peptides exert multiple cardioprotective and homeostatic effects, predominantly mediated via guanylate cyclase-A–coupled receptors, including promotion of natriuresis and diuresis, inhibition of the SNS and the RAAS, as well as antifibrotic actions on myocardial tissue [34]. Figure 7 summarizes the mechanisms underlying natriuretic peptide production and their role in cirrhosis. A major limitation to their clinical applicability is the significant variability in circulating levels observed in conditions such as advanced age, obesity, and ascites, as well as their limited specificity, since elevated natriuretic peptide concentrations are encountered in both cardiovascular and non-cardiovascular disorders, including ischemic stroke, renal dysfunction, and liver disease [35]. In the context of liver cirrhosis, natriuretic peptides have demonstrated utility as prognostic biomarkers [36] and as indicators of cirrhotic cardiomyopathy in patients undergoing liver transplantation [31,32,37].

Although interpretation can be limited by age, obesity, ascites, renal dysfunction, and other non-cardiac diseases, natriuretic peptides remain valuable for prognosis and prediction of CCM in liver transplant candidates [36].

Plasma NT-proBNP levels are often elevated in cirrhosis and correlate with disease severity, as assessed by the Child–Pugh classification [33].

In our study, we have demonstrated that NT-proBNP levels had a weak correlation with diastolic dysfunction. Given that NT-proBNP levels can also be influenced by hepatic impairment, we performed a multimarker evaluation to obtain a probability score for diastolic dysfunction using the most sensitive combination of clinical and biochemical parameters.

Considering the limitations of natriuretic peptides, recent attention has shifted to sST2, a member of the interleukin-1 receptor superfamily that acts as a decoy receptor for interleukin-33 [38].

Interleukin 33 (IL-33) normally exerts cardioprotective effects against hypertrophy and fibrosis in the stressed myocardium, but elevated sST2 levels blunt this protection [39]. Studies have demonstrated that in chronic HF, sST2 predicts outcomes independently of NT-proBNP and high-sensitivity troponin T [40].

sST2 has emerged as a valuable prognostic biomarker in chronic heart failure. Unlike NT-proBNP, sST2 is largely independent of several confounding factors, including renal dysfunction, age, body mass index, and atrial rhythm disturbances. Elevated circulating sST2 levels reflect myocardial stress, inflammation, and fibrosis, and have been consistently associated with increased mortality, higher rates of hospitalization, and adverse cardiovascular outcomes. These characteristics make sST2 a complementary biomarker to natriuretic peptides, providing additional prognostic information beyond conventional markers, particularly in conditions where NT-proBNP interpretation may be limited [41].

Its role in cirrhosis remains under investigation, but it may offer complementary prognostic value for detecting CCM. In the multimarker model, sST2, NT-proBNP, and age emerged as independent predictors of diastolic dysfunction.

A multimarker assessment in patients with cirrhosis is essential not only for identifying diastolic dysfunction, but also for guiding phenotype-oriented management of cirrhotic cardiomyopathy, where therapeutic strategies differ between systolic and diastolic phenotypes.

#### 2.2.2. Treatment of Cirrhotic Cardiomyopathy: Current Strategies and Biomarker-Based Implications

To date, no disease-specific therapy exists for cirrhotic cardiomyopathy (CCM). Management, therefore, remains largely supportive and is usually initiated only after the development of overt heart failure. Therapeutic strategies are primarily extrapolated from conventional heart failure management and focus on preload and afterload reduction. However, this approach is particularly challenging in patients with cirrhosis, given the presence of systemic vasodilatation, reduced effective arterial blood volume, and a baseline tendency toward hypotension, all of which may limit the tolerability of standard heart failure therapies [42].

Diuretics remain essential for symptomatic management, particularly in patients with fluid overload and ascites, but require careful titration to avoid worsening renal perfusion. Mineralocorticoid receptor antagonists are commonly used and may exert beneficial effects beyond volume control by reducing myocardial fibrosis and neurohormonal activation. In contrast, angiotensin-converting enzyme inhibitors and angiotensin receptor blockers are generally poorly tolerated in advanced cirrhosis due to their vasodilatory effects and the risk of precipitating renal dysfunction, and are therefore often contraindicated.

The use of angiotensin receptor–neprilysin inhibitors (ARNI) in CCM is highly limited. Owing to hepatic metabolism, potential drug accumulation, hypotension, and renal hypoperfusion, ARNI therapy may only be considered in carefully selected patients with compensated cirrhosis (Child–Pugh A), preserved renal function, and under close cardiologic and hepatologic monitoring. These agents should be avoided in decompensated cirrhosis.

Non-selective beta-blockers (NSBBs) are a cornerstone therapy in heart failure with reduced ejection fraction and are widely used in cirrhosis for the prevention of variceal bleeding and rebleeding [43]. Despite their extensive use, robust evidence supporting their efficacy in CCM is currently lacking. This is particularly relevant given that CCM is characterized by impaired chronotropic and inotropic responses to physiological and pharmacological stress. From a pathophysiological perspective, NSBBs may exert divergent effects in CCM, potentially improving electrophysiological abnormalities such as QT prolongation, while simultaneously exacerbating chronotropic incompetence and hypotension.

In this context, we believe that prospective clinical trials investigating the relationship between NSBB therapy and multimarker assessment—including NT-proBNP and soluble ST2 (sST2)—could provide valuable insights into disease progression, myocardial stress, and treatment response in CCM patients.

In patients with cirrhotic cardiomyopathy and elevated NT-proBNP, therapeutic possibilities include interventions targeted at reducing cardiac wall stress—such as optimized diuretic therapy, Inhibitors of sodium–glucose cotransporter 2 (SGLT2) inhibitors, and natriuretic-peptide–enhancing strategies—as well as emerging agents modulating hemodynamics and preventing progression to systolic dysfunction. SGLT2 has demonstrated cardiovascular and renal benefits in heart failure and chronic kidney disease, and is being explored in cirrhosis. Their natriuretic, anti-inflammatory, and renal-protective effects may theoretically improve cardio–hepato–renal dysfunction in CCM, particularly in patients with refractory ascites. However, safety concerns—including hypotension, renal dysfunction, and euglycemic ketoacidosis—limit their use in advanced cirrhosis, and robust clinical data are still lacking [44,45].

The elevation of sST2, reflecting activation of the IL-33/ST2 pathway and ongoing myocardial fibrosis, supports therapeutic exploration of molecules, such as antifibrotic agents (pirfenidone, nintedanib), anti-inflammatory modulators (IL-1β inhibitors), and experimental strategies targeting ST2 expression or enhancing IL-33 cardioprotective signaling, including monoclonal anti-sST2 antibodies, IL-33 agonists, and therapies designed to suppress endothelial sST2 production [45].

Increasing evidence suggests that systemic inflammation, oxidative stress, and mitochondrial dysfunction play central roles in the pathogenesis of CCM. Accordingly, therapeutic strategies targeting these mechanisms have gained interest.

Niaz et al. [46] investigated the effects of atorvastatin in a bile duct ligation (BDL) rat model of cirrhotic cardiomyopathy. Atria from BDL rats exhibited a blunted chronotropic response to isoproterenol compared with sham-operated controls, whereas atorvastatin treatment restored this responsiveness. In addition, atorvastatin significantly reduced serum levels of BNP, TNF-α, and malondialdehyde, supporting the hypothesis that statins may improve cardiac dysfunction in CCM through anti-inflammatory and antioxidant mechanisms.

Taurine, an abundant amino sulfonic acid with antioxidant, anti-inflammatory, and mitochondrial-stabilizing properties, represents another promising therapeutic agent. In cirrhosis, impaired hepatic synthesis leads to reduced myocardial taurine levels and weakened antioxidant defenses. Experimental models of CCM demonstrate that taurine supplementation attenuates oxidative stress, enhances mitochondrial ATP production, and reduces myocardial injury markers, thereby improving cardiac performance [45].

Similarly, Sheibani et al. [47] demonstrated that spermidine administration in a BDL rat model significantly improved cardiac contractility and electrocardiographic parameters. These functional improvements were accompanied by marked reductions in inflammatory mediators (TNF-α, IL-1β), oxidative stress markers (malondialdehyde), and NF-κB expression, along with increased antioxidant defenses, such as superoxide dismutase and glutathione. These findings highlight spermidine as a potential modulator of inflammation-driven myocardial dysfunction in CCM.

Albumin therapy has emerged as a clinically relevant adjunctive treatment in patients with decompensated cirrhosis. Beyond its role in volume expansion, albumin exerts pleiotropic effects, including binding of endotoxins, reduction in oxidative stress, and modulation of inflammatory pathways. Fernandez et al. [48] conducted a prospective clinical study evaluating the effects of high-dose albumin administration (1.5 g/kg weekly for 12 weeks) in patients with decompensated cirrhosis. Albumin therapy normalized serum albumin levels improved systemic hemodynamics and left ventricular function, and significantly reduced circulating pro-inflammatory cytokines, without significantly affecting portal pressure. These findings suggest that albumin may attenuate cardiocirculatory dysfunction and systemic inflammation in cirrhosis, thereby indirectly improving CCM-related cardiac impairment.

Experimental targets such as liver X receptor (LXR) agonists and galectin-3 inhibitors are also being explored, given their potential to modulate inflammation, myocardial fibrosis, and adverse remodeling, but remain investigational at present [45].

An ongoing prospective clinical trial evaluates the therapeutic proprieties of colchicine in HFpEF by targeting the systemic inflammation that plays a key pathophysiological role in heart failure (using hsCRP and sST2 as biological markers) and its effects on the levels of N-terminal pro–B-type natriuretic peptide, indices of left ventricular diastolic function and remodeling, measures of right ventricular systolic function, and left atrial volumetric characteristics [49].

## 3. Materials and Methods

### 3.1. Study Population

We conducted a prospective, observational, analytical case–control group study lasting one year. Eighty-three patients hospitalized in the Gastroenterology and Internal Medicine Departments of an Emergency Clinical County hospital (ECCH) for 12 months (1 June 2020–1 July 2021) were included in the study, with the approval of the Bioethics Commission of “Iuliu Hațieganu” University of Medicine and Pharmacy in Cluj-Napoca (No. 413/14 November 2018). All patients included in the study provided written consent to participate [50].

### 3.2. Inclusion Criteria: Patients Were Divided into Two Groups

(1) Patients with non-alcoholic liver cirrhosis: this group comprised 43 patients with cirrhosis hospitalized in the Internal Medicine I and Gastroenterology Departments of ECCH Cluj. Inclusion criteria were a confirmed diagnosis of non-alcoholic liver cirrhosis in accordance with current clinical guidelines [1], age ≥ 18 years, good understanding of the Romanian language, and provision of informed consent to participate in the study.

(2) Control group: The control group consisted of 40 asymptomatic individuals evaluated in the outpatient setting of the Internal Medicine I or Cardiology I Departments of ECCH Cluj.

### 3.3. Exclusion Criteria

Children (<18 years old), known/newly diagnosed structural heart diseases (valvulopathies, cardiomyopathies, congenital heart diseases), atrial fibrillation, systolic dysfunction, collagen diseases, known lung/ kidney disease or hepatocellular carcinoma; patients taking cardiotoxic, vasoactive or nitrate medication; patients who undergone radiotherapy; gastrointestinal bleeding within the last 3 months patients suffering from an active bacterial infection (inflammation based on high-sensitivity C-reactive protein [hs-CRP] ≥ 10 mg/dL).

### 3.4. Study Protocol

All participants underwent a standardized evaluation that included clinical assessment, laboratory investigations, electrocardiography, transthoracic echocardiography, and abdominal ultrasonography.

For laboratory tests, venous blood samples were obtained under sterile conditions with minimal venous stasis. Biochemical measurements were performed using a Konelab 30 I analyzer (Thermo Scientific, Waltham, MA, USA). The assessed parameters included blood glucose, high-density lipoprotein (HDL) cholesterol, low-density lipoprotein (LDL) cholesterol, uric acid, triglycerides, hs-CRP, creatinine clearance (mL/min) and corrected creatinine clearance (mL/min/1.73 m^2^), serum albumin, total bilirubin, hepatic transaminases, and international normalized ratio (INR).

Serum samples were subsequently stored at −80 °C and later analyzed for NT-proBNP and sST2 concentrations using sandwich enzyme-linked immunosorbent assay (ELISA) techniques.

All samples were analyzed in duplicate in accordance with the manufacturer’s instructions. NT-proBNP levels were measured using an Elabscience Biotechnology kit (Houston, TX, USA; catalog number E-EL-H6126; detection range: 0.16–10 ng/mL; sensitivity: 0.09 ng/mL), while sST2 levels were determined using an Elabscience Biotechnology kit (Houston, TX, USA; catalog number EH3822; detection range: 0.156–10 ng/mL; sensitivity: 0.094 ng/mL).

For each analyte, a calibration curve was generated using the protein standards supplied with the manufacturer’s recommendations. Absorbance was measured using a ClarioStar microplate reader (BMG Labtech, Ortenberg, Germany), and data acquisition and analysis were conducted with the integrated MARS 3.1 software. A four-parameter logistic calibration model was used for the quantification of the final concentrations, which were expressed as the mean of the duplicate measurements.

Child Scoring classifies patients suffering from liver cirrhosis to determine the severity of the disease based on five clinical and laboratory criteria: serum bilirubin, serum albumin, INR, encephalopathy, and ascites, in three classes: Child A (5–6 points), Child B (7–9 points) and Child C (10–15 points) [1].

Electrocardiograms were performed upon admission.

A cardiac ultrasound examination was performed for each patient using a phased-array (sector probe), optimized for intercostal cardiac imaging with an adult cardiac transducer 3SP frequency range: 1.6–5.5 MHz on a GE HealthCare LOGIQ S7 (GE HealthCare Technologies, Inc., General Electric Company, Boston, MA, USA) ultrasound machine to evaluate left ventricular (LV) structural and performance parameters. Standard and Tissue Doppler Imaging (TDI) echocardiography were used according to the 2016 ASE/EACVI standards [51] to determine the systolic function of the left ventricle by measuring left ventricle ejection fraction (LVEF) from the apical 4-chamber view. LA and LV dimensions, and the thicknesses of the LV septum and posterior wall, were determined from the parasternal long-axis view. Pulsed wave (PW) Doppler was used for assessing trans-mitral flow at the tips of the mitral leaflets, and continuous wave (CW) Doppler was used for evaluating tricuspid regurgitant systolic jet velocity in the apical four-chamber view. Fractional area change (FAC) and TAPSE were measured to determine RV systolic function. In the apical four-chamber view, PW TDI was used at the level of the septal and lateral mitral annulus to obtain the following characteristics: maximum diastolic velocity during the early filling phase at the septal and lateral mitral annulus (e′) and average E/e′ velocities.

Abdominal ultrasonography was performed in all patients using a GE HealthCare LOGIQ S7 (GE HealthCare Technologies, Inc., General Electric Company, USA), equipped with a convex transducer (C1-5) operating at 1.8–5 MHz. The participants were examined in the supine position after fasting for at least 6 h. The evaluation included the liver, intrahepatic and extrahepatic bile ducts, gallbladder, pancreas, spleen, kidneys, and major abdominal vessels. For each patient, the following parameters were assessed: liver size, echostructure, surface contour, presence and amount of ascites, and diameters of the portal and splenic veins.

### 3.5. Statistical Method

Data analysis was conducted using Python (version 3.11) with relevant statistical libraries, including pandas for data manipulation, stats models for logistic regression, and scikit-learn for model development and performance evaluation. Descriptive statistics were calculated to provide an overview of the data set, and Mann–Whitney U tests were used to compare patient and control groups for NT-proBNP and sST2 levels. Feature importance was performed using Random Forest models, and Youden’s J-index was employed to determine optimal cut-off points. Decision tree models were iteratively refined based on predictor importance and data availability to maximize predictive accuracy while minimizing data loss. Receiver Operating Characteristic (ROC) curves were generated to evaluate the discriminatory power of the final model, with Area Under the Curve (AUC) values serving as key performance indicators.

## 4. Conclusions

The combined assessment of NT-proBNP and sST2 improves the early diagnostic accuracy for detecting diastolic dysfunction in patients with cirrhotic cardiomyopathy by integrating complementary pathophysiological pathways—NT-proBNP reflecting congestion and hemodynamic stress, and sST2 reflecting inflammation and myocardial remodeling. A multi-marker approach enhances diagnostic performance compared with each biomarker alone and supports clinical decision-making in cirrhotic cardiomyopathy. The concomitant elevation of both biomarkers may have therapeutic implications, particularly for strategies aimed at reducing cardiac wall stress and targeting inflammatory or fibrotic pathways.

### Study Limitations

This study has several limitations. First, the relatively small, single-center sample restricts generalizability and limits statistical power, especially when comparing Child–Pugh subgroups. Although echocardiographic evaluation followed current guidelines, image acquisition can be technically difficult in patients with cirrhosis, and more advanced modalities, such as GLS or cardiac MRI, were not included. The presence of various comorbidities may also have influenced the results. Although efforts were made to account for some of these confounding factors in the statistical analysis, the complexity and interdependence of these variables limit the ability to completely disentangle their individual effects on biomarker concentrations. Future studies should seek to address these limitations by enrolling larger, multicenter cohorts, more thoroughly adjusting for concomitant therapies and comorbid conditions, and employing longitudinal study designs to better elucidate causal relationships.

## Figures and Tables

**Table 1:** Comparison between clinical characteristics and lab test results in the analyzed groups.

Patient Characteristics	Study Lot (n = 43)	Control Lot (n = 40)	*p*
Sex (M/F)	19/24	22/18	NS
Age (Years)	62.66+/−7.55	57.6+/−12.75	NS
BMI (kg/mp^2^)	28.72+/−3.67	29.39+/−3.25	NS
Scor Child-Pugh			
Child A Class (n%)	30 (76.35)		
Child B Class (n%)	9 (13.15)		
Child C Class (n%)	4 (10.5)		
Heart rate (b/min)	73+/−11	55+/−12	NS
Ascites	11 (25.6%)	-	
Splenomegaly	19 (44%)	-	
Encefalopathy	4 (9.3%)	-	
Total Bilirubin (mg/dL)	1.39 ± 1.11		
Creatinine (mg/dL)	0.80 ± 0.33		
Albumin (g/dL)	3.72 ± 0.68		
NT-proBNP (pg/mL)	94.17 ± 151	19.2 ± 5.47	*p* < 0.001
sST2 (ng/mL)	5.4 ± 2.31	2.4 ± 0.99	*p* < 0.001

BMI—Body Mass Index; NS—Not significant; NT-proBNP—N-terminal pro–B-type Natriuretic Peptide; sST2—soluble suppression of tumorigenicity 2. Data are presented as mean ± SD or number of patients expressed as percentages.

**Table 2:** Comparative echocardiographic and electrocardiographic characteristics in patients with cirrhosis versus controls.

Ultrasound Parameters	Study Lot (n = 43 pts)	Control Lot (40)	*p*
E wave (m/s)	0.67 ± 0.23	0.65 ± 0.19	NS
A wave (m/s)	0.69 ± 0.21	0.72 ± 0.17	NS
E/A	0.9 ± 0.3	0.85 ± 0.43	NS
EDT (ms)	251 ± 60	245 ± 42.7	NS
Lateral e′ ± velocity (cm/s)	10 ± 3.3	11 ± 1.4	NS
Septal e′ velocity (cm/s)	8.3 ± 2.8	8.8 ± 2.9	NS
E/e′ > 14	4 (10.52%)	0	
LAVI (mL/mp)	40	35	*p* < 0.05
VmaxSTr > 2.8 m/sec	6 (15.78%)	0	
DD	22 (51%)	22 (55%)	NS
LVDD grade 1	15 (34.8%)	22 (55%)	
LVDD grade 2	6 (15.78%)	0	
LVDD grade 3	1 (2.63%)	0	
ECG Parameters			
QTc (ms)	444.987+/−94.214	422.811+/−30.192	NS

E wave—early diastolic transmitral flow velocity; A wave—late diastolic (atrial) transmitral flow velocity; E/A ratio—ratio between early and late diastolic filling velocities; EDT—E-wave deceleration time; Lateral e′ velocity—early diastolic mitral annular velocity measured at the lateral annulus; Septal e′ velocity—early diastolic mitral annular velocity measured at the septal annulus; E/e′ > 14—marker of elevated left ventricular filling pressure; LAVI—left atrial volume index; DD—diastolic dysfunction, VmaxSTr > 2.8 m/s—peak tricuspid regurgitation velocity greater than 2.8 m/s; LVDD—left ventricular diastolic dysfunction (grades 1–3); QTc—corrected QT interval. Data are presented as mean ± SD or number of patients expressed as percentages.

## Data Availability

The original contributions presented in this study are included in the article. Further inquiries can be directed to the corresponding author.

## Abbreviations

The following abbreviations are used in this manuscript

ADH	Antidiuretic Hormone
ARNI	Angiotensin Receptor–Neprilysin Inhibitors
AUC	Area Under the Curve
BDL	Bile Duct Ligation
BMI	Body Mass Index
BNP	B-type Natriuretic Peptide
CCM	Cirrhotic Cardiomyopathy
cGMP	Cyclic Guanosine Monophosphate
CO	Carbon Monoxide
CW	Continuous Wave (Doppler)
E	Early transmitral flow velocity
ECCH	Emergency Clinical County Hospital
ECG	Electrocardiogram
e′	Early diastolic mitral annular velocity
ELISA	Enzyme-Linked Immunosorbent Assay
FAC	Fractional Area Change
FLIP	Flice-like Inhibitory Protein
GLS	Global Longitudinal Strain
HDL	High-Density Lipoprotein
HF	Heart Failure
hs-CRP	High-sensitivity C-Reactive Protein
IL-33	Interleukin 33
INR	International Normalized Ratio
LA	Left Atrium/Left Atrial
LAVI	Left Atrial Volume Index
LDL	Low-Density Lipoprotein
LV	Left Ventricle/Left Ventricular
LVDD	Left Ventricular Diastolic Dysfunction
LVEF	Left Ventricular Ejection Fraction
LVSD	Left Ventricular Systolic Dysfunction
MHC	Myosin Heavy Chain
MR-proANP	Mid-Regional Pro-Atrial Natriuretic Peptide
MRI	Magnetic Resonance Imaging
NO	Nitric Oxide
NSBBs	Non-selective beta-blockers
NT-proBNP	N-terminal pro–B-type Natriuretic Peptide
PARP	Poly(ADP-ribose) Polymerase
PW	Pulsed Wave (Doppler)
RAAS	Renin–Angiotensin–Aldosterone System
ROC	Receiver Operating Characteristic
RV	Right Ventricle/Right Ventricular
SD	Standard Deviation
SNS	Sympathetic Nervous System
sST2	Soluble Suppression of Tumorigenicity 2
TAPSE	Tricuspid Annular Plane Systolic Excursion
TDI	Tissue Doppler Imaging
TNF-α	Tumor Necrosis Factor Alpha
TR	Tricuspid Regurgitation
Vmax TR	Maximal Systolic Velocity of Tricuspid Regurgitation
